# Interventions at the Transition from Prison to the Community for Prisoners with Mental Illness: A Systematic Review

**DOI:** 10.1007/s10488-018-0848-z

**Published:** 2018-01-23

**Authors:** G. Hopkin, S. Evans-Lacko, A. Forrester, J. Shaw, G. Thornicroft

**Affiliations:** 1 Department of Health Policy, London School of Economics and Political Science, London, UK; 2 Centre for Implementation Science, Health Service and Population Research Department, Institute of Psychiatry, Psychology and Neuroscience, King’s College London, London, UK; 3 Personal Social Services Research Unit, London School of Economics and Political Science, London, UK; 4 Offender Health Research Network, University of Manchester, Manchester, UK

**Keywords:** Prison, Mental health, Transition, Community mental health, Systematic review

## Abstract

Prisoners have high rates of mental illness and the transition from prison to the community is a problematic time for the provision of mental health services and a range of negative outcomes have been identified in this period. A systematic review was conducted to identify interventions for prisoners with diagnosed mental health conditions that targeted this transition period. Fourteen papers from 13 research studies were included. The interventions identified in this review were targeted at different stages of release from prison and their content differed, ranging from Medicaid enrolment schemes to assertive community treatment. It was found that insurance coverage, and contact with mental health and other services can be improved by interventions in this period but the impact on reoffending and reincarceration is complex and interventions may lead to increased return to prison. There is a developing evidence base that suggests targeting this period can improve contact with community mental health and other health services but further high quality evidence with comparable outcomes is needed to provide more definitive conclusions. The impact of programmes on return to prison should be evaluated further to establish the effect of interventions on clinical outcomes and to clarify the role of interventions on reincarceration.

**Electronic supplementary material:**

The online version of this article (10.1007/s10488-018-0848-z) contains supplementary material, which is available to authorized users.

## Introduction

It is well established that prisoners have high rates of mental health problems compared to the general population (Fazel and Danesh 2002; Fazel and Seewald 2012). Prison mental health services are increasingly being developed to identify and treat those with diagnosed mental health conditions during their time in custody. However, the transition from prison to the community is stressful for prisoners with mental health problems and their families and a range of negative outcomes have been identified in this period.

Continuity of care between prison and community-based health services is difficult to provide, and prisoners often lose contact with services after release. Prisoners are unlikely to be registered with primary care services which represents a barrier to care (Social Exclusion Unit 2002) and even for prisoners with severe mental illness, contact with community mental health care is rare in the months after release (Hamilton and Belenko 2015; Lennox et al. 2012; Ventura et al. 1998) and the care that they receive does not reflect the need indicated by their complex and comorbid conditions (Begun et al. 2015). This lack of planned contact may also lead to an increase in chaotic and unplanned interactions with health services after release (Fox et al. 2014; Mallik-Kane and Visher 2008) and increased emergency department utilisation for problems related to mental health (Frank et al. 2013).

In addition to lack of contact with health services, a number of other serious negative outcomes have been identified in this period. All-cause mortality for prisoners after release from prison is higher than in the general population (Farrell and Marsden 2008) and the risk of suicide for released prisoners is high in the first month in the community (Pratt et al. 2006). In both cases, having a diagnosed mental health condition confers additional risk (Lize et al. 2015). Prisoners with severe mental illness may also have poor outcomes on forensic measures with higher rates of reoffending and return to prison, especially in those with co-occurring substance use disorders (Baillargeon et al. 2010).

The aim of this systematic review is to identify interventions aimed at improving outcomes in the transition from prison to the community for prisoners diagnosed with a mental health condition and to review their efficacy on health insurance coverage, health service use and forensic outcomes. Other systematic reviews and meta analyses have looked at mental health interventions implemented during other stages of the Criminal Justice System (Kouyoumdjian et al. 2015; Martin et al. 2012) but this is the first systematic review to focus on the transition from prison to the community which represents a time in the pathway to care for this population which is amenable to improvement.

## Method

### Search Strategy

The following electronic databases were searched in January 2017: PsycInfo, Medline, EMBASE, CINAHL, CENTRAL, ASSIA, BNI, Criminal Justice, OpenGrey, BASE Search. A common set of search terms relating to population, setting, transition period and design was used in each database, as well as subject headings specific to each database (Online Appendix I). The Boolean operators “AND” and “OR” were used to combine terms. No limits were set with regards to year of publication or country of origin. Experts in the field were identified from studies included in the initial search and from the authors’ knowledge and were contacted. Reference lists of relevant systematic reviews were reviewed for additional articles. The systematic review was not registered before completion but a predefined protocol was followed.

### Inclusion Criteria

A screening tool was specified in advance and articles were considered eligible for inclusion if they met all of the following criteria: Participants were detained in a prison facility, were diagnosed with a mental health condition and had been released to the community; and the intervention was focused on the transition from prison to the community. Interventions based on any treatment model were included and could be provided pre or post-release period or both, as were interventions that were not based on health outcomes (e.g. housing and employment support). Randomised and non-randomised trials were included, and due to lack of research in this area so were trials with no comparison group. Articles were not excluded based on their country of origin and articles that were not in English were included if a translated version could be accessed.

### Study Selection, Data Extraction and Synthesis

After the databases had been searched, two reviewers screened 20% of the titles and abstracts that remained after removal of duplicates and a high level of agreement was found (> 95%), one reviewer (GH) proceeded with screening of the remaining results. The full reports of potentially relevant studies were retrieved and all studies that met the inclusion criteria were included. One author (GH) then extracted data for all included studies using a pre-piloted form. Information was extracted on study characteristics, participant characteristics, and the effect of interventions on outcomes in the transition from prison to the community. High levels of expected heterogeneity meant that a narrative synthesis was conducted.

### Quality Assessment

The Effective Public Health Practice Project Quality Assessment Tool for Quantitative Studies (Effective Public Health Practice Project 2009) was used to determine the quality of the included studies’ methodology. The tool allows quality assessment for randomised and non-randomised methods and assesses studies on the following elements of bias: selection bias, design, confounders, blinding, data collection methods, withdrawals and drop outs. Studies are rated strong if all elements are rated as strong or moderate, moderate if one element is rated as weak, or weak if two or more elements are rated as weak.

## Results

### Search Results

**Fig. 1:**
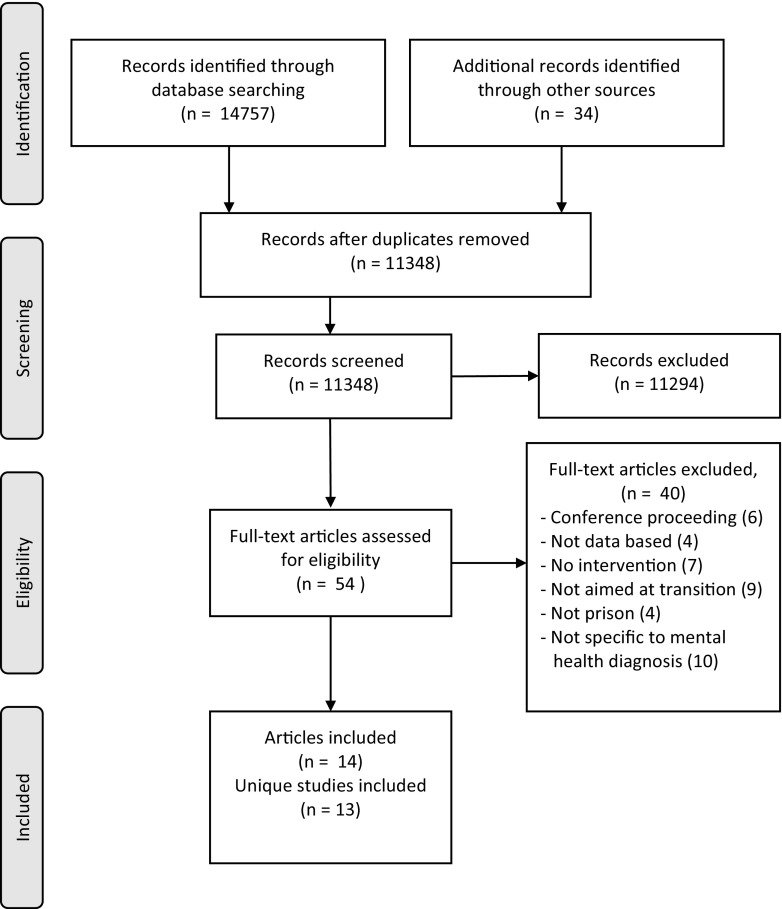
PRISMA flowchart (Moher et al. 2009)

A total of 14,757 articles were identified from the search and a further 34 articles were located from expert recommendations and reference checking. After removal of duplicates, 11,348 articles were screened according to inclusion criteria and the full texts of 54 articles were retrieved to make a final decision on eligibility. Fourteen articles were found to be eligible for inclusion and, as two referred to the same study (Brown et al. 2013; Buck et al. 2011), the included articles concerned 13 research studies and data was extracted from each. This method adhered to the principles outlined in the PRISMA statement (Moher et al. 2009) (Fig. 1).

### Characteristics of Included Studies

The majority of the included studies were conducted in the United States of America (US; n = 10; Brown et al. 2013; Buck et al. 2011; Burke and Keaton 2004; Hartwell and Orr 1999; Kesten et al. 2012; Morrissey et al. 2016; Roskes and Feldman 1999; Solomon and Draine 1995; Theurer and Lovell 2008; Trupin et al. 2011; Wenzlow et al. 2011) with two studies conducted in England (Jarrett et al. 2012; Shaw et al. 2017) and one in Australia (Green et al. 2016). Nine used a wide geographical area which included urban and rural settings, whereas four related to a single urban area (Brown et al. 2013; Buck et al. 2011; Roskes and Feldman 1999; Solomon and Draine 1995; Theurer and Lovell 2008). Most of the studies used adult samples (n = 12) and one used a sample of juvenile offenders (Trupin et al. 2011). None of the studies were restricted to a single disorder and criteria for inclusion in the studies ranged from solely being diagnosed with a mental health condition, being treated by a mental health team within the prison, being adjudged to be of high risk or in distress, and being homeless before entry into custody.

Six studies were based on cohort comparisons (Green et al. 2016; Kesten et al. 2012; Morrissey et al. 2016; Theurer and Lovell 2008; Trupin et al. 2011; Wenzlow et al. 2011), either from facilities that did not offer the intervention, from a time when the intervention was not available, or with a group not referred to an intervention. Four studies were randomised controlled trials (Burke and Keaton 2004; Jarrett et al. 2012; Shaw et al. 2017; Solomon and Draine 1995). Two were case series with no comparison group (Hartwell and Orr 1999; Roskes and Feldman 1999) and one used a pre-post comparison with outcomes compared to a comparable time period for the same individuals before contact with the program (Brown et al. 2013; Buck et al. 2011). Study outcomes ranged from contact with health services, Medicaid enrolment, reoffending and reincarceration, sanctions for treatment non-compliance and place of residence at time of treatment discharge.

Most of the studies were bridging interventions, with intervention provided both before and after release, but there were also examples of care being provided only during the pre (n = 2) or post (n = 2) release period. The majority of the interventions were delivered by health services and used a mixed approach (n = 8) which incorporated multiple interventions including case management, psychosocial modules and onward referral. Two studies focused on Medicaid enrolment and two relied on specialist mental health staff embedded in probation teams and working alongside corrections staff. Several of the programmes provided help with issues surrounding drug use but none of the interventions included this as their primary goal. A wide range of health professionals and corrections staff were involved in the delivery of interventions, as well as in one case supervised students studying for a Masters in Psychology. More details on the interventions are shown in Table 1.

**Table 1:** Intervention details

Reference	Stage of intervention	Description of intervention	Length of delivery	Professional involved	Provider/funder
Brown et al. (2013) Buck et al. (2011)	Pre and post-release	Case management services provided to obtain appropriate medical and psychiatric care and housing. Daytime release and an escort to the local health centre arranged	Not reported	Clinician. Qualifications not reported	Homeless for Houston; Harris County Mental Health Authority
Burke and Keaton (2004)	Pre and post-release	Specially trained probation staff provided support with housing, employment, family relations and financial planning as well as linkage to health services. As outside agencies became more involved the Connections team reduced their support	Targeted 4–6 contacts prior to release and up to 12 months after release	Social worker, probation officer, psychiatrist	California Board of Corrections; San Diego County Sheriff’s Dept.; San Diego County Probation Dept
Green et al. (2016)	Pre and post-release	*TCP*: full time transition clinicians support medical staff coordinated mental health release planning before and after release with the aim of providing continuity *TR*: individually tailored social and recovery support was available and assistance was given on goal-setting, accommodation, life skills, finances, linkage to services and recreational activities	Pre-release not reported *TCP*: up to 2 weeks after release *TR*: up to 6 months after release	*TCP*: nursing and allied health professionals *TR*: support worker with psychology and social work background	Queensland Health; Richmond Fellowship Queensland
Hartwell and Orr (1999)	Pre and post-release	Program staff considered information on psychosocial and criminal variables and formulate a plan for release. Staff continue to provide case coordination and consultation after release	Up to 3 months before and 3 months after release	Qualifications not reported	Massachusetts Department of Corrections
Jarrett et al. (2012)	Pre and post-release	A CTI manager identified barriers to engagement and provides support and case management before and after release to facilitate contact in the community	Up to 4 weeks before and 6 weeks after release	Mental health professional (i.e. nurse, psychologist, psychiatrist)	Medical Research Council; Psychiatric Research Trust; Oxleas NHS Trust
Morrissey et al. (2016)	Pre-release	Staff identified eligible individuals and invited them to apply for expedited Medicaid enrolment and assisted with this. Applicants were required to appear for review at community services offices after release	Not reported	Corrections mental health staff	Washington State Legislature
Kesten et al. (2012)	Pre and post-release	Prisoners completed the life skills re-entry curriculum which focused on managing emotions and life skills. After release therapists stayed in contact until links had been made with community services	Life skills re-entry for 9–12 months before release Follow up period not reported	Psychologist, social worker or other experienced professional	Conn. Dept. of Correction; Conn. Dept. of Mental Health and Addiction Services; US Dept. Of Justice
Roskes and Feldman (1999)	Post-release	The team provided medical treatment, case management, psychosocial services and illicit drug use monitoring. Similar to a CMHT model	Until probation conditions were lifted	Psychiatrist, MSc level therapist, probation officer	Baltimore City Probation Office
Shaw et al. (2017)	Pre and post-release	A CTI manager identified barriers to engagement and provides support and case management before and after release to facilitate contact in the community	Up to 4 weeks before and 6 weeks after release	Mental health professional (i.e. nurse, psychologist, psychiatrist)	National Institute of Health Research, local NHS Mental Health Trusts
Solomon and Draine (1995)	Post-release	*ACT*: prisoners were assigned to a local ACT team who provided training in community living, assertive outreach and advocacy *FC*: prisoners were assigned to a forensic caseworker who brokered services in the community teams they were based	*ACT*: 1 year after release *FC*: unlimited	ACT: psychiatrist, mental health nurse, housing specialist FC: mental health nurse	National Institute of Mental Health; Philadelphia Mental Health Agency
Theurer and Lovell (2008)	Pre and post-release	The team conducted a pre-release assessment and made a treatment plan for after release. After release intensive case management was provided along with 24 h crisis support. The team closely coordinate with community correction officers. For part of the study, the intervention included voluntary confinement to a residential site	Up to 3 months before release Follow up period not reported	Mental health nurse, psychiatrist, substance abuse counsellor, housing manager, community corrections officer	Washington State Department Of Social and Health Services Washington State Department of Corrections
Trupin et al. (2011)	Pre and post-release	Coaches delivered a manualised intervention based on multi-systemic and dialectical behaviour therapy and motivational enhancement. A parent skills training module was also available	Up to 3 months before release and 6 months after release	MSc level clinician, PhD level consultant, psychiatrist	Washington State Legislature
Wenzlow et al. (2011)	Pre-release	A discharge manager based in the Department of Correction identified prisoners with SMI and arranged Medicaid enrolment for day of release and assisted with federal benefit applications	Up to 4 months before release	Not reported	Oklahoma State Mental Health Agency

### Outcomes

#### Health Insurance Coverage

Two studies aimed to ensure that prisoners with mental illness were enrolled in Medicaid at re-entry to facilitate access to health services by reducing financial barriers (Morrissey et al. 2016; Wenzlow et al. 2011). In an Oklahoma state based study (Wenzlow et al. 2011), participants in the intervention group had higher rates of Medicaid enrolment on the day of re-entry (25%) compared with those at the facility before the introduction of the intervention (8%) and comparison facilities without the intervention (3%). When enrolment at entry and other appropriate variables were controlled for, there was a significant difference in enrolment on the day of release (*p* = .012) and after 90 days (*p* = .008).

Morrissey, Domino and Cuddeback (2016) evaluated a similar initiative in Washington state using more robust methods. Prisoners who were referred to the expedited Medicaid program in the early years of the initiative were compared to a similar group of prisoners who were not referred due to limits in the capacity of the program as it was rolled out. In order to control for differences in the groups, propensity weighted models were used to account for a wide range of baseline variables. Medicaid enrolment for participants in the intervention group was significantly higher at release by 35 weighted percentage points (pp), as well as at 30 day and 12 month follow up time points (all *p* < .01).

#### Health Service Use and Clinical Outcomes

Both studies examining expedited Medicaid enrolment found a beneficial effect on health service contact. In Wenzlow et al. (2011), the study’s secondary outcomes were significant with more of the intervention group having contact with mental health services (*p* = .009) and being prescribed medication (*p* = .041) in the 90 days following release. Morrissey et al. (2016) also found participants in the intervention group also had higher rates of mental health and other health service use as well as prescribed medication and although this information was recorded from insurance based payment systems the differences appear robust.

Theurer and Lovell (2008) compared prisoners in the Washington State Mentally Ill Offender Community Transition Program (MIOCTP) with a matched sample of prisoners from earlier studies. They found that those in the MIOCTP group had an average of 2.3 days to contact with mental health services compared to 185 days in the matched control group and had more hours of contact with mental health staff both in prison (20 vs. 0.7 h) and the community (25 vs. 2.5 h). Significance levels were not reported for these outcomes. In addition, two articles reporting the same study from Houston, Texas (Brown et al. 2013; Buck et al. 2011) suggest that daytime release followed by escort to a health care centre and case management significantly improved linkage with health services (*p* < .001).

In the three studies from outside of the US, Jarrett et al. (2012) evaluated the critical time intervention (CTI) in a pilot randomised controlled trial in English prisons. A large drop out limited the validity of the results due to the study lacking sufficient power to detect a difference, but a higher proportion of CTI participants had positive outcomes on most outcomes and they were significantly more likely to be registered with a general practitioner (87 vs. 38%; *p* = .01) and be receiving medication (80 vs. 38%; *p* = .03). With the feasibility of the CTI demonstrated in the pilot but power lacking, Shaw et al. (2017) conducted a larger randomised controlled trial with adapted research methods, which included recruiting a larger sample and seeking to reduce drop out after randomisation by using an algorithm to predict whether prisoners awaiting trial or sentencing would be released within the time frame of the study and collecting data from routinely collected sources. For the primary outcome, it was found that participants in the CTI arm had significantly improved engagement, as measured by evidence of a care coordinator, evidence of a care plan and evidence of medical treatment, with community mental health teams at 6 weeks (53 vs. 27%, *p* = .012) and this was maintained at a later follow up 6 months (*p* = .029) after release. At 6 weeks after release, participants in the CTI arm also had significantly higher levels of registration with GPs (*p* = .018). In Australia, Green et al. (2016) found that those than had long term support from Transition Reintegration, Recovery and Support (TR) were significantly more likely to be in contact with mental health services than those who received a shorter time with TR support or only standard transition arrangements by the prison mental health team (*p* < .001).

Only a single study examined clinical and psychosocial outcomes. This randomised controlled trial compared two interventions, assertive community treatment and forensic caseworkers, with treatment as usual but did not find a significant difference in these outcomes (Solomon and Draine 1995).

#### Forensic Outcomes

In terms of reoffending, in the two articles reporting on the same sample (Brown et al. 2013; Buck et al. 2011), it was found that prisoners with SMI who were expected to be homeless on release and received intervention were less likely to commit felonies (*p* < .001) or misdemeanours (*p* < .001) and were less likely to be booked (*p* < .001) or charged (*p* < .001) for offences than in the 6 months prior to entry to custody. Kesten et al. (2012) compared prisoners referred to Connecticut Offender Re-entry Program (CORP) to standard treatment planning. A lower proportion of those in the CORP group were rearrested within 3 months (9.1 vs. 15.6%) and a lower proportion was also arrested in the following 3–6 months (4.5 vs. 12.6%) but these differences were not significant. Similarly, in the study by Theurer and Lovell (2008) it was found that those in the MIOCTP had lower levels of recidivism for felony (23 vs. 42%; *p* = .01) and other offences (39 vs. 61%; *p* = .003). For juvenile offenders in Washington state, Trupin et al. (2011) found that a family based integration programme was associated with lower felony recidivism (*p* < .05) but this was not the case for overall, violent felony or misdemeanour recidivism.

Expedited Medicaid enrolment was not associated with a reduction in arrests and participants in the intervention arm had higher levels of incarceration in jail (13% points, *p* < .01) or state prisons (7% points, *p* < .01) than those who followed the usual process (Morrissey et al. 2016). Similarly, Solomon and Draine (1995) found, in opposition to their hypothesis, that more participants in assertive community treatment (ACT; 60%) returned to prison than those with a forensic caseworker (FC; 40%) or in usual services (36%) although this difference was reported as not significant. Green et al. (2016) also examined reincarceration and found that participants in the long term support group had higher 50% survival in the community but this trend was not consistent at further time points. This is likely due to lack of randomisation and is influenced by reasons for referral to modes of care other than the long term support group.

In the only study to find significant results in both reoffending and reincarceration, Burke and Keaton (2004) evaluated a corrections based intervention for prisoners with mental illness and low functioning prior to release. They reported that participants who received the Connections intervention were less likely to be booked into jail for a new offence during the year follow up than the comparison group (35 vs. 46%, p < .05) and also spent less total days in jail as a result of new offences and parole revocation (34.6 vs. 20.2 days; p < .01). When the group was analysed with only participants who completed the Connections programme this difference was more pronounced.

Roskes and Feldman (1999) found that three out of 16 patients received criminal sanction for treatment non-compliance, compared to nine of 16 who had had sanctions previously and Hartwell and Orr (1999) examined the effect of a forensic transition team and found that at discharge after three months 57% of patients remained in the community, 23% were hospitalised and 10% were reincarcerated. In both cases an appropriate comparison group was not included and the effect of the interventions cannot be assessed with these findings.

#### Quality of Included Studies

Seven studies were rated weak (Brown et al. 2013; Buck et al. 2011; Burke and Keaton 2004; Green et al. 2016; Hartwell and Orr 1999; Jarrett et al. 2012; Roskes and Feldman 1999; Solomon and Draine 1995) according to the Quality Assessment Tool (Effective Public Health Practice Project 2009), four were rated moderate (Shaw et al. 2017; Theurer and Lovell 2008; Trupin et al. 2011; Wenzlow et al. 2011) and two were rated strong (Kesten et al. 2012; Morrissey et al. 2016). Details of the quality assessment are given in Online Appendix II. Blinding was a particular issue for the included studies with outcome assessors knowing intervention status and participants aware of the aims of the study.

## Discussion

This systematic review found 14 articles relating to 13 studies of interventions aimed at the transition from prison to the community for individuals with mental health problems. The results of these studies suggest that interventions aimed at the transition from prison to the community can improve health insurance coverage and increase contacts with mental health and other health services and this approach should be pursued more widely. However, this systematic review reveals some concerning trends regarding return to custody after involvement with interventions aimed at transition and the impact of interventions on reoffending is not clear. The primary outcome of the majority of the included studies was based on forensic outcomes, such as lowering recidivism rates, and whilst this is an important area, a key rationale for interventions aimed at this period is to prevent severe negative health outcomes of prisoners with mental illness after release (Baillargeon et al. 2010; Lize et al. 2015). Despite this rationale, only one study evaluated the impact of interventions on behavioural and clinical outcomes and no studies examined all-cause or drug related mortality, or suicide and more emphasis is needed to establish whether interventions do have an effect on these important outcomes.

Two studies of expedited Medicaid enrolment conducted in different US states show that significant improvements in enrolment on release can be made, that these differences are sustained over time and that this is associated with increased use of mental health services (Morrissey et al. 2016; Wenzlow et al. 2011). Many US states have adopted Medicaid enrolment initiatives for released prisoners, however, 16 state prison systems still have no provision for Medicaid enrolment at release and this should be addressed. The issue of insurance coverage is not present in countries with tax based universal health care systems but it is notable that this review found no similar interventions addressing insurance coverage rates in other countries with private or social insurance. In these countries, coverage may be terminated on entry to prison and enrolment is not automatic on release and this issue should be examined.

With regards to health service use, both studies of expedited Medicaid enrolment found that the intervention group had higher levels of contact with mental health services (Morrissey et al. 2016; Wenzlow et al. 2011) and in one, there were increased numbers of prescriptions for psychiatric medication but there were also increased rates of emergency care use (Morrissey et al. 2016). When case management interventions were considered and health outcomes were reported, it was found that contact with mental health services could be increased (Green et al. 2016; Theurer and Lovell 2008), as could primary care registration and receipt of medication in England (Jarrett et al. 2012).

Although studies have shown improvements in contact with mental health and other health services, it appears possible that interventions aimed at improving health outcomes in transition have a negative impact on return to prison after release. Solomon and Draine (1995) and Morrissey et al. (2016) both found that participants in the arm which was aimed at improving mental health outcomes had higher rates of reincarceration. This was despite other studies suggesting that rates of offending were reduced for some types of crimes (Brown et al. 2013; Buck et al. 2011; Kesten et al. 2012; Trupin et al. 2011). One study which examined the Connections intervention was able to examine both offending and return to custody and reported both lower rates of offending and fewer returns to custody in the intervention group, as well as fewer overall days spent in custody (Burke and Keaton 2004).

Given the negative impact of returning to prison for those with mental health problems, it is important to consider how this could be avoided and the study by Burke and Keaton highlighted above (2004) may point to solutions. Their Connections intervention was different from other interventions included in this review as probation workers worked alongside mental health staff and receive training on mental health awareness and alternative options to parole revocation for people with mental health problems. It is possible that contact with services increases monitoring, including drug testing, and this greater awareness leads to increased parole violations and higher rates of parole revocation, unless probation staff are involved in the delivery of the intervention and are provided with alternatives to reincarceration as they are in Burke and Keaton’s (2004) Connections intervention. This notion is supported by evidence that specialised mental health probation services lead to increased awareness of the difficulties prisoners with mental illness face and can promote the use of strategies other than parole revocation where violations occur (Wolff et al. 2014). This issue of increased return to prison is certainly worth monitoring and additional studies that examine both reoffending and reincarceration are needed to draw more definitive conclusions.

Whilst research in this area is not yet well developed, there are a number of recommendations that can be made to clinicians and administrators about the design of interventions that target the transition period. All of the interventions, apart from the unsuccessful trial by Solomon and Draine (1995) and low quality Roskes and Feldman (1999) paper, begin while a prisoner was still detained and involved planning for release through referrals to community health and other social services and where required, enrolment in health insurance prior to the expected date of release. This pre-release component appears important and means that attempts to ensure continuity of care are arranged at the earliest possible opportunity. Planning in the pre-release period should be seen as the minimum requirement for interventions but where services are already developed or more resources are available, it appears important that support is also provided in the post-release period to complement prior efforts. Prisoners report that they have difficulties in arranging their own care after release due to lack of knowledge of services and how to engage with them and sending referrals prior to release may not be sufficient to ensure that continuity is realised (Binswanger et al. 2011) meaning that support is the post-release period will be beneficial. This post-release support take the form of remote follow up or more involved contacts with released prisoners but should be focused on ensuring that prisoners are reminded of and prompted to attend appointments and should also involve follow up with community services to ensure that referrals have been processed and actions are being taken to ensure continuity of care.

In addition, it has been noted above that interventions to improve outcomes during transition may increase return to prison for this group. The exact reasons for this are unclear and further research is needed to examine this issue but in the interim, health services and health professionals working in this area or developing interventions should ensure that they develop links with local probation services and consider other ways of reducing the impact of increased monitoring that may occur when contact with health services is made.

### Limitations

An extensive list of search terms was used and a number of databases were searched but it is possible that more data is available on this question which we were not able to identify with our search strategy. The included studies are limited to English speaking countries and a recent textbook on international prison psychiatry was reviewed in an attempt to identify additional interventions. No interventions that would have been eligible were cited in chapters on a wide range of countries even though transition to the community was frequently mentioned (Konrad et al. 2013).

In addition, the inclusion criteria required prisoners to be diagnosed with a mental health condition and this meant that interventions which recruited prisoners with only substance abuse problems alongside those with diagnosed mental health conditions would not have been included. Several of the interventions in the included studies did provide some focus on drug use but targeting substance abuse was not the primary aim of any intervention and this is an important gap, given the prevalence of drug use in the period immediately after release and the additional risk conferred by having a comorbid mental health problems and substance misuse. The approach taken in this review may have excluded drug use based interventions which have been trialled with samples of the wider prison population but importantly their impact on this specific group with particular additional needs has not been proven.

### Future Directions

Meta-analytic methods were not possible in this review due to the heterogeneity of the methods and interventions of included studies. The methods of future studies will inevitably differ due to local considerations and availability of data but researchers should consider using equivalent health and forensic outcomes and follow up periods which would allow more comprehensive comparison. This more coordinated approach would help to answer questions about comparative effectiveness of different approaches including whether pre-release, post-release, or combined pre-post release interventions are most effective and whether inclusion of particular professional groups (i.e. mental health staff, social workers, and probation staff) is particularly important.

In addition, the included studies were mostly of weak or of moderate quality and few high quality studies have been conducted with this population. Several studies used randomised methods, demonstrating their feasibility in this setting and this approach should be replicated more widely. If experimental methods are not possible, especially where a change in policy has taken place, it is important for researchers to use the highest quality methods possible and the propensity score matching used by Morrissey et al. (2016) is a good example of how confounders can be controlled for in the absence of randomisation. This could be replicated in future studies. Blinding was not present or not reported in a number of studies and whilst the aims of interventions are transparent to participants, more attempts should be made to blind researchers to trial arms.

## Conclusion

There is an emerging body of evidence that interventions for prisoners with mental illness aimed at the transition from prison to the community can improve health insurance coverage and contact with mental health and other health services. The evidence for a reduction in reoffending is equivocal with small improvements and non-significant results found but there is also a concerning trend that these interventions could increase reincarceration through increased monitoring. Further high quality trials are needed to examine these outcomes in more detail and there should be efforts to design and report trials to allow more comprehensive comparison. The majority of existing studies are based in the US and more trials are also needed across the world to ensure the findings are replicable in differing prison and health systems.

## Electronic Supplementary Material

Below is the link to the electronic supplementary material.

Supplementary material 1 (DOCX 18 KB)
