# Supplementary material for: Interventions at the Transition from Prison to the Community for Prisoners with Mental Illness: A Systematic Review
Source: Adm Policy Ment Health. 2018 Jan 23;45(4):623–34. doi: 10.1007/s10488-018-0848-z (PMC5999162; doi:10.1007/s10488-018-0848-z)
Supplement: Supplementary file 1 — Supplementary material 1 (DOCX 18 KB) [file 10488_2018_848_MOESM1_ESM.docx]

**Appendix I.**

**Search Strategy**

*Population Terms (Mental Illness):*

“mental*”

“severe mental illness”

“SMI”

“severe and persistent mental illness”

“serious mental illness”

“schizo*”

“depressi*”

“bipolar”

“psychiat*”

“psychos*”

“psychot*”

“patient*”

“service user*”

“client*”

“diagnos*”

*Population Terms (Prisoners):*

“prison*”

”*offend*”

“remand*”

“sentence*”

“*detain*”

“*criminal*”

“*convict*”

“*felon*”

“(pre- OR pre OR under- OR under) trial”

“jail*”

“gaol*”

“detention”

“correction*”

“forensic”

*Population Terms (Released):*

“re-entry OR reentry”

“release*”

“reintegrat* OR re-integrat*”

“transition”

“supervis*”

“discharg*”

“bail*”

“probation*”

“parole*

“resettle*”

*Design Terms:*

“(randomised OR randomized) controlled trial”

“(randomised OR randomized) control trial”

“(randomised OR randomized) clinical trial”

“random*”

“control*”

“RCT”

“pragmatic trial”

“controlled trial”

“control trial”

“proof of (concept OR principle) trial”

“control group”

“quasi*”

“quasi experiment*”

“non-randomised OR non randomised OR non-randomized OR non randomized”

“cohort study”

“case-control OR case control”

“case-series OR case series”

“pilot”

“prospective”

“longitudinal”

*Intervention Terms:*

“program*”

“programme*”

“treat*”

“therap*”

“(case OR care) management”

“(case OR care) AND (coordination OR co-ordination)”

“psychosocial OR psycho-social”

“intervention*”

“discharge plan*”

“care plan*”

“(pre OR pre-) discharge”

“(post OR post-) discharge”

“community”

**Database Specific Subject Headings**

**OVID SEARCHES**

**PSYCINFO**

*Population Terms (Mental Illness):*

exp Psychiatric Patients/

exp Mental Disorders/

exp Mental Health/

*Population Terms (Prisoners):*

exp Prisoners/

exp Prison/

exp Mentally Ill Offenders/

*Population Terms (Released):*

exp Criminal Rehabilitation/

exp Parole/

exp Probation/

exp Institutional Release/

exp Facility Discharge/

*Design Terms:*

exp Clinical Trials/

exp Treatment Effectiveness Evaluation/

exp Mental Health Program Evaluation/

*Intervention Terms:*

exp Treatment/

exp Discharge Planning/

exp Case Management/

exp Mental Health Services/

**EMBASE**

*Population Terms (Mental Illness):*

exp Mental Patient/

exp Mental Disease/

exp Mental Health/

*Population Terms (Prisoners):*

exp Prison/

exp Prisoner/

*Population Terms (Released):*

exp Rehabilitation/

exp Community Reintegration/

*Design Terms:*

exp Controlled Clinical Trial/

exp Clinical Trial/

exp Controlled Study/

exp Randomized Controlled Trial/

*Intervention Terms:*

exp Psychiatric Treatment/

exp Prison Nursing/

exp Patient Care Planning/

exp Mental Health Service/

**MEDLINE**

*Population Terms (Mental Illness):*

exp Mental Disorders/

exp Mental Health/

exp Mentally Ill Persons/

*Population Terms (Prisoners):*

exp Prisoners/

exp Prison/

*Population Terms (Released):*

NO APPROPRIATE

*Design Terms:*

Exp Clinical Trial/

Exp Treatment Outcome/

*Intervention Terms:*

Exp Therapeutics/

Exp Patient Care Planning/

Exp Mental Health Services/

**EBSCOHOST SEARCHES**

**CINAHL**

*Population Terms (Mental Illness):*

(MH “Psychiatric Patients+”)

(MH “Mental Disorders+”)

*Population Terms (Prisoners):*

(MH “Mentally Ill Offenders”)

(MH “Correctional Facilities”)

(MH “Prisoners”)

*Population Terms (Released):*

(MH “Probation”)

*Design Terms:*

(MH “Clinical Trials+”)

(MH “Randomized Controlled Trials”)

*Intervention Terms:*

(MH “Community Health Services+”)

(MH “Patient Care+”)

**PROQUEST SEARCHES**

**British Nursing Index**

*Population Terms (Mental Illness):*

SU.EXACT.EXPLODE("Psychiatric Disorders")

SU.EXACT.EXPLODE("Mental Health")

SU.EXACT.EXPLODE("Psychiatric Patients")

*Population Terms (Prisoners):*

SU.EXACT.EXPLODE("Prison Nursing")

SU.EXACT.EXPLODE("Prison Health Services")

SU.EXACT.EXPLODE("Mentally Disordered Offenders")

*Population Terms (Released):*

NO APPROPRIATE

*Design Terms:*

SU.EXACT.EXPLODE("Research Methods")

*Intervention Terms:*

**SU.EXACT.EXPLODE("Community Psychiatric Nursing") SU.EXACT.EXPLODE("1:Mental Health ") SU.EXACT.EXPLODE("Community Health Services")**

**SU.EXACT.EXPLODE("Community Care")**

**ASSIA**

*Population Terms (Mental Illness):*

SU.EXACT.EXPLODE("Psychiatric Disorders")

SU.EXACT.EXPLODE("Mental Health")

*Population Terms (Prisoners):*

SU.EXACT.EXPLODE("Prisons")

SU.EXACT.EXPLODE("Prisoners")

SU.EXACT.EXPLODE("Violent Mentally Ill People")

*Population Terms (Released):*

SU.EXACT.EXPLODE("Release")

SU.EXACT.EXPLODE("Parole")

*Design Terms:*

SU.EXACT.EXPLODE("Randomized Controlled Trials ")

*Intervention Terms:*

**SU.EXACT.EXPLODE("Community Psychiatric Nursing") SU.EXACT.EXPLODE("Community Health Services")**

**SU.EXACT.EXPLODE("Community Care")**

**Criminal Justice**

Population Terms (Mental Illness):

SU.EXACT.EXPLODE("Mental Disorders")

SU.EXACT.EXPLODE("Mental Health")

Population Terms (Prisoners):

SU.EXACT.EXPLODE("Prisons")

SU.EXACT.EXPLODE("Prisoners")

Population Terms (Released):

SU.EXACT.EXPLODE("Rehabilitation of Criminals")

SU.EXACT.EXPLODE("Parole & Probation")

Design Terms:

SU.EXACT.EXPLODE("Clinical Trials ")

Intervention Terms:

**SU.EXACT.EXPLODE("Psychiatric – Mental Health Nursing") SU.EXACT.EXPLODE("Community Health Care")**

**SU.EXACT.EXPLODE("Correctional Treatment Programs")**

LIMIT - Title and Abstract

**COCHRANE SEARCHES**

**CENTRAL**

Population Terms (Mental Illness):

[Mental Disorders] explode all trees

[Mentally Ill Persons] explode all trees

Population Terms (Prisoners):

[Prisons] explode all trees

[Prisoners] explode all trees

Population Terms (Released):

NO APPROPRIATE

Design Terms:

[Clinical Trial] explode all trees

Intervention Terms:

[Mental Health Services] explode all trees

LIMIT - In Trials

**Search Strategy for Grey Literature Databases**

BASE Search

(mental* OR severe mental illness OR SMI OR severe persistent mental illness OR serious mental illness OR schzio* OR depressi* OR bipolar* OR psychiat* OR psychos* OR psychot* OR patient* OR service user* OR client* OR diagnos*) AND (prison* OR offend* OR remand* OR sentence* OR detain* OR criminal* OR convict* OR felon* OR jail* OR gaol* OR detention* OR correction* OR forensic*) AND (reentry OR re-entry OR release* OR reintegrat* OR re-integrat* OR transition OR supervis* OR discharg* OR bail* OR probation* OR parole* OR resettle) AND (program* OR treat* OR therap* OR intervention*)

LIMIT – Reports, Papers, Lectures

OpenGrey

(mental* OR severe mental illness OR SMI OR severe persistent mental illness OR serious mental illness OR schzio* OR depressi* OR bipolar* OR psychiat* OR psychos* OR psychot* OR patient* OR service user* OR client* OR diagnos*) AND (prison* OR offend* OR remand* OR sentence* OR detain* OR criminal* OR convict* OR felon* OR jail* OR gaol* OR detention* OR correction* OR forensic*) AND (reentry OR re-entry OR release* OR reintegrat* OR re-integrat* OR transition OR supervis* OR discharg* OR bail* OR probation* OR parole* OR resettle) AND (randomi?ed controlled trial OR randomi?ed control trial OR randomi?ed clinical trial OR random* OR control* OR RCT OR pragmatic trial OR controlled trial OR control trial OR proof of concept trial OR proof of principle trial OR control group OR quasi* OR cohort study OR case control OR case-control OR case series OR case-series OR pilot OR prospective OR longitudinal) AND (program* OR treat* OR therap* OR intervention*)

**Appendix II.**

**Quality Assessment**

EPHPP Quality Assessment Tool Ratings for Studies Included in the Systematic Review.

|  | Overall | Selection Bias | Study Design | Confounders | Blinding | Data Collection Methods | Drop Out |
| --- | --- | --- | --- | --- | --- | --- | --- |
| Solomon & Draine (1995) | **WEAK** | 2 | 1 | 3 | 2 | 2 | 3 |
| Hartwell & Orr (1999) | **WEAK** | 2 | 3 | 3 | 2 | 2 | 3 |
| Roskes & Feldman (1999) | **WEAK** | 3 | 3 | 3 | 2 | 2 | 2 |
| Burke & Keaton (2004) | **WEAK** | 3 | 1 | 3 | 2 | 3 | 3 |
| Jarrett et al. (2012) | **WEAK** | 2 | 1 | 1 | 3 | 2 | 3 |
| Brown et al. (2013) Buck et al. (2011) | **WEAK** | 3 | 2 | 3 | 2 | 3 | 2 |
| Green et al. (2016) | **WEAK** | 3 | 2 | 3 | 2 | 2 | 2 |
| Theurer & Lovell (2008) | **MODERATE** | 2 | 2 | 1 | 3 | 2 | 2 |
| Trupin et al. (2011) | **MODERATE** | 2 | 2 | 1 | 3 | 2 | 2 |
| Wenzlow et al. (2011) | **MODERATE** | 2 | 2 | 1 | 3 | 2 | 2 |
| Shaw et al. (2017) | **MODERATE** | 2 | 1 | 1 | 3 | 2 | 2 |
| Kesten et al. (2011) | **STRONG** | 1 | 2 | 1 | 2 | 2 | 2 |
| Morrissey et al. (2016) | **STRONG** | 1 | 2 | 1 | 2 | 2 | 2 |

1 = Strong, 2 = Moderate, 3 = Weak.
